# Play Active physical activity policy intervention and implementation support in early childhood education and care: results from a pragmatic cluster randomised trial

**DOI:** 10.1186/s12966-023-01442-0

**Published:** 2023-04-20

**Authors:** Emma K. Adams, Andrea Nathan, Stewart G. Trost, Jasper Schipperijn, Trevor Shilton, Georgina Trapp, Clover Maitland, Ashleigh Thornton, Matthew Mclaughlin, Phoebe George, Elizabeth Wenden, Hayley Christian

**Affiliations:** 1 Telethon Kids Institute, University of Western Australia, 35 Stirling Hwy, Perth, 6009 WA Australia; 2 School of Population and Global Health, University of Western Australia, 35 Stirling Hwy, Perth, 6009 WA Australia; 3 School of Human Movement and Nutrition Sciences, University of Queensland, Brisbane, 4072 QLD Australia; 4 Department of Sports Science Clinical Biomechanics, University of Southern Denmark, Campusvej 55, Odense, 5230 Denmark; 5 School of Human Sciences, The University of Western Australia, 35 Stirling Hwy, Perth, 6009 WA Australia; 6 Centre for Behavioural Research in Cancer, Cancer Council Victoria, 615 St Kilda Rd, Melbourne, 3004 VIC Australia

**Keywords:** Physical activity, Childcare, Policy, Intervention, Implementation, Preschool

## Abstract

**Background:**

Policy interventions to increase physical activity in early childhood education and care (ECEC) services are effective in increasing physical activity among young children. However, a large proportion of ECEC services do not have nor implement a physical activity policy. Play Active is an evidence-informed physical activity policy intervention with implementation support strategies to enable ECEC services to successfully implement their policy. This study examined the effectiveness, implementation, and process outcomes of Play Active.

**Methods:**

A pragmatic cluster randomised trial in 81 ECEC services in Perth, Western Australia was conducted in 2021. Services implemented their physical activity policy over a minimum of three months. The effectiveness outcomes were changes in educator practices related to daily time provided for total physical activity and energetic play. Implementation outcomes included changes in director- and educator-reported uptake of policy practices and director-reported uptake of high impact and low effort policy practices. Process evaluation outcomes included awareness, fidelity, reach, and acceptability of the intervention and implementation strategies. Analysis involved descriptive statistics and generalised linear mixed effects models.

**Results:**

There was a significant increase in the uptake of director-reported policy practices (*p* = 0.034), but no change in the uptake of the subset of high impact and low effort policy practices. Intervention group educators reported high awareness of the Play Active policy recommendations (90%). Play Active acceptability was high among educators (83%) and directors (78%). Fidelity and reach were high for most implementation support strategies (> 75%). There were no significant changes in the amount of physical activity or energetic play educators provided to children or in the proportion of educators providing the policy recommended ≥ 180 min of physical activity/day or ≥ 30 min of energetic play/day for intervention compared to wait-listed comparison services.

**Conclusions:**

Play Active resulted in significantly higher uptake of physical activity practices. However, there was no change in the amount of physical activity provided to children, which may be explained by the relatively short policy implementation period. Importantly, Play Active had high awareness, fidelity, reach, and acceptability. Future research should investigate the effectiveness of Play Active over longer implementation periods and its scalability potential.

**Trial registration:**

Australian New Zealand Clinical Trials Registry (reference number 12620001206910, registered 13/11/2020, https://www.anzctr.org.au/Trial/Registration/TrialReview.aspx?id=378304&isReview=true).

**Supplementary Information:**

The online version contains supplementary material available at 10.1186/s12966-023-01442-0.

## Background

Establishing positive physical activity behaviours early in life is vital for young children’s health and development [1]. Physical activity supports young children’s cardiorespiratory and musculoskeletal systems, helps maintain a healthy weight status, and enables positive mental health and social-emotional and cognitive development [1]. However, a large proportion of young children do not meet the recommended 180 min of physical activity per day including 60 min of energetic play [2, 3] as outlined in national and World Health Organisation (WHO) 24-Hour Movement Guidelines for the Early Years [4, 5].

Outside of the home, many young children spend a considerable amount of time each week in early childhood education and care (ECEC). Internationally, approximately 25% of children aged 0 to 2 years and 90% of children aged 3 to 5 years are enrolled in formal ECEC [6]. In Australia, over 40% of children aged 0 to 5 use childcare and attend for an average 25 h per week [7]. Previous research suggests ECEC services have a greater influence on young children’s physical activity than socio-demographic factors [8]. Thus, ECEC is an important physical activity intervention setting able to reach many young children [9].

In line with the socio-ecological model of behaviour change, interventions that combine individual and environmental (including policy) level factors are more effective than interventions focused on single-level factors [10]. An evidence-based ECEC physical activity policy intervention is an effective strategy to improve educators’ physical activity provision, and children’s physical activity levels [11–14]. Yet, in Australia and other countries such as the United States and Canada, less than half of ECEC services have a written physical activity policy [14, 15]. There is also considerable variation within countries such as Australia. For example, 58% of ECEC services in New South Wales [16] but only 16% of ECEC services in Western Australia [17] have physical activity-related statements in their service policies, despite national standards requiring services to support and promote children’s physical activity. The Australian Children’s Education & Care Quality Authority (ACECQA) National Quality Framework for Early Childhood Education and Care states that ‘Each child’s health and physical activity is supported and promoted’ [18]. However, while these national standards exist, there is no specific information on how much physical activity children should do whilst attending care, or resources and training to support educators to provide children in their care with the daily physical activity they need.

Simply having a physical activity policy is insufficient for supporting adequate physical activity levels. An ECEC-specific physical activity policy is more likely to be effective if implemented in conjunction with effective implementation supports such as educator professional development and training [19]. Furthermore, policy implementation needs to account for the local and broader ECEC implementation context and barriers and enablers, including committed and practical leadership, organisation and educator capacity related to funding and staffing, educator mindset related to perceived weather barriers and risk aversion, and levels of parent engagement [14, 20]. Intervention research is required to understand the impact of ECEC-specific physical activity policies and implementation strategies on educator’s physical activity provision-related practices [19, 21].

To address these needs, Play Active, a physical activity policy intervention with supporting implementation strategies, was developed. The central component of Play Active is an evidence-informed physical activity policy template containing 25 practices to support nine age-specific recommendations and two key statements which provide clear guidance on the amount of physical activity and sedentary time, including screen time, young children should do while attending ECEC [22]. There are six implementation support strategies to facilitate policy implementation within ECEC services, including policy personalization, policy review and approval, resource guide, brief assessment tool, professional development, and Project Officer implementation support [22].

This study examined the effectiveness of the Play Active intervention on changes in educator-reported daily time provided for children to be physically active at ECEC. The uptake of the policy practices by directors and educators (implementation outcomes), and the fidelity, reach, awareness, and acceptability of Play Active (process outcomes) were also examined.

## Methods

The trial protocol has previously been described in full [22]. The Consolidated Standards of Reporting Trials (CONSORT) checklist [23] with cluster [24] and pragmatic [25] extensions and the Template for Intervention Description and Replication (TIDieR) checklist [26] are provided in Additional File 1 and Additional File 2, respectively. Ethics approval was obtained from The University of Western Australia Human Research Ethics Committee (RA/4/20/6120 approved 19/5/2020).

### Trial design and setting

Play Active was evaluated using a pragmatic cluster randomised trial design, defined as a randomised controlled trial in which pre-existing groups or clusters (ECECs) are randomly allocated to the treatment arms, and whose purpose is to inform decisions about practice [25]. It was chosen to test the Play Active physical activity policy intervention and accompanying implementation support strategies with ECEC service staff (directors and educators), while simultaneously enabling the program to be as close to real world conditions as possible [22, 25]. The trial was undertaken in 2021 and involved 81 long-day ECEC services in Perth, Western Australia. At the time of recruitment, there were a total of 557 long-day ECEC services in the study area. In Australia, long-day ECEC services offer care and education for children prior to compulsory schooling. ECEC services are highly regulated through the ACECQA National Quality Framework, which includes assessing and rating ECEC services on seven quality areas in the National Quality Standards, including children’s health and safety [27]. Based on ECEC sector advice, the trial was completed within a calendar year to minimise the impact of staffing and child enrolment changes within ECEC services. There were no changes to trial methods or outcomes after trial commencement.

### Participants and recruitment

ECEC services were recruited via an ‘Expression of Interest’ form available on a study partner’s website and contacted by study project officers to determine eligibility. Ineligibility criteria included: services catering exclusively to children requiring specialist care, mobile preschools, Department of Education and Communities preschools, services already involved in another trial, and services with or expecting a significant management change in the last/next three months. Eligible service directors were provided with study information and provided consent for their service to participate. Directors provided contact details for eligible (full- or part-time) educators; the research team then invited these educators to participate (consent included as part of the educator survey). Recruitment of educators was ongoing throughout the trial.

### Sample size

The trial required a minimum of 60 ECEC services and 309 educators at post-intervention to detect a 15-min change in educator total time provided for children’s indoor and outdoor physical activity (80% power, 2-tailed alpha level of 0.05, ICC of 0.01–0.05, and assuming 30% educator dropout).

### Randomization and blinding

At the conclusion of baseline data collection, 81 ECEC services were randomly allocated to either the intervention (*n* = 41) or wait-listed comparison (*n* = 40) groups using a central randomisation procedure. The randomisation sequence was generated using a computerised random number function in Microsoft Excel. To avoid contamination between services, services of the same provider in close geographical proximity to each other were randomly allocated to the same group. The research team member generating the allocation sequence and assigning services to their group was not involved with recruitment, data collection, or intervention delivery. Due to the nature of the Play Active intervention it was impossible to blind services to their group allocation.

### Play Active intervention

Play Active was developed to be a low-cost, feasible, acceptable, and high-fidelity program [22]. The central component of Play Active was a four-page editable evidence-informed physical activity policy template [19] which was emailed to intervention ECEC service directors after they completed baseline data collection (April-June 2021). The policy template included two overarching key statements and nine age-specific recommendations on the amount of physical activity and sedentary time (including screen time), for young children whilst in ECEC. To support achieving these recommendations, the policy template also included 25 physical activity-related practices specific to management and educators (*n* = 14), the physical environment (*n* = 4), parent and carer engagement (*n* = 5), and policy monitoring and review (*n* = 2) (Additional File 3). Seven of these practices were rated ‘high impact and low effort’ during the policy development process [19]. To facilitate policy implementation in ECEC services, six implementation support strategies were provided: (i) policy personalisation; (ii) policy review and approval; (iii) resource guide; (iv) brief energetic play assessment tool; (v) educator physical activity professional development and training; and (vi) project officer implementation support [22] (Table 1).

**Table 1:** Description of Play Active strategies and process measures for intervention services

Intervention	Mode of delivery	Dose and timeframe	Description	Process measures and results		
				Fidelity^a^	Reach^a^	Acceptability
Physical activity policy	Email and word document	One email as part of group allocation notification	ECEC service directors were sent the policy document when being notified of their group allocation	100% of services were sent the policy	At post-intervention, 75% of educators knew their service had a policy and 99% of these knew where to find their policy	83% of educators and 78% of directors were satisfied or very satisfied with Play Active. 58% of directors found Play Active very useful or extremely useful. Directors also reported their educators found Play Active useful (75%), were willing to engage with Play Active (85%), understood the policy recommendations (83%), were confident to apply the recommendations (75%), were enthusiastic about Play Active (63%)^b^
Implementation support strategies						
(i) Personalise policy	Email and phone	After receiving the policy document, services were contacted weekly to a maximum of 2 months to remind them to personalise their policy and return it for review and approval	ECEC directors were asked to personalise their policy template to suit their service and then return a copy to Play Active team. Services were asked to select a minimum of 5 of 25 practices to prioritise during the implementation period. ECEC services were encouraged to select their five practices from seven ‘high impact and low effort’ practices identified in previous testing [19]	Overall, 100%: 95% of intervention services were contacted for policy personalisation and review and two services were not contacted as they returned their policy prior to the planned first contact	100% of services personalised the policy. Services took a mean of 32 days to return their policy (min 7, max 62, median 27, IQR 26)	N/A
(ii) Policy review & approval	Email	Review/approval process completed within one week of receipt of personalised policy. Approved policy document sent back to directors	Two Play Active project officers independently reviewed and approved policies. A third project officer resolved any concerns or disputes The minimum approval criteria were the personalised policy contained: two (out of two) key statements; nine (out of nine) recommendations; and at least five (out of 25) practices	100% of policies were reviewed and approved	100% of services selected at least five practices	N/A
(iii) Resource guide	Post and email	After policy approval, services were mailed a hard copy resource guide. A PDF copy was provided upon request via email	The resource guide was evidence-informed and included practical strategies educators could use to implement each of the 25 practices in the physical activity policy, evidence-informed explanations of what each procedure means, and signposting to various evidence-based resources for more information	100% of services were sent a hard copy resource guide	54% of educators reported they had used the resource guide. Of these, 4% used it once, 4% used it less than once a month, 51% used it a few times each month, 21% used it once a week, 14% used it more than once a week, and 7% used it daily	68% of educators who reported using the resource guide found it very useful or extremely useful
(iv) Brief energetic play assessment tool	Post and email	Printed copy provided via mail. PDF available upon request via email. Included as part of baseline and post-intervention data collection	Educators were encouraged to use the brief energetic play assessment tool to monitor and assess children’s physically activity whilst at ECEC	100% of services were provided with the energetic play assessment tool as part of the resource guide and as part of data collection	80% of intervention services completed the energetic play assessment tool at baseline. 75% of intervention services completed the energetic play assessment tool at post-intervention	N/A
(v) Professional development	Email and partner websites	Provider 1: Six online training modules (30–60 min each). Individual educators completed at own pace. Provider 2: Five service-level self-paced e-learning modules (3–4 h each), including up to 40 segments of content. Estimated six weeks to complete	Two study partners provided the professional development (Provider 1 and Provider 2). Training was designed to upskill educators in providing more physical activity opportunities for children in their care, including specific skills on developing fundamental movement skills and active play-based learning. Provider 1 training was provided to services for free as part of the trial. Provider 2 training was offered as part of the trial at a discounted cost of $99 per module (5 modules total) per service	100% of intervention services were provided with welcome emails from the providers and access to the professional development	*Educator level:* 39% of educators self-reported they had used the Provider 1 training. Provider 1 website data showed only 11% of educators registered and enrolled in the course and only 5% completed the training. 13% of educators self-reported using the Provider 2 training. *Service level:* 30% of services had at least 1 educator complete the Provider 1 training. No services completed the Provider 2 paid e-learning modules	84% of educators who reported using Provider 1 training felt the training was very useful or extremely useful; 12% felt it was moderately useful. 85% of educators who reported using Provider 2 training felt the training was very useful or extremely useful
(vi) Project officer implementation support	Phone	Mid-implementation (1.5–2 months) phone call to directors, maximum three phone call attempts	A mid-implementation ‘check-in’ with service directors to determine whether policy implementation had commenced, discuss any challenges they were facing, and check they had received the resources and professional development providers welcome emails	100% of intervention services were attempted to be contacted at mid-implementation	88% of services provided information on their implementation of Play Active at the mid-implementation check in	N/A

*Notes*: The term ‘policy’ refers to the Play Active physical activity policy template. Fidelity is operationalised as whether the intervention was provided to services as intended in the protocol [22]. Reach is operationalised as the uptake of the intervention within services by directors and educators. Acceptability is operationalised as educator- or director-reported usefulness of and/or satisfaction with the intervention

^a^Based on 40 intervention ECEC services; one service who was randomised to the intervention group is excluded from counts as they withdrew prior to being able to receive the intervention

^b^Response of agree or strongly agree

Directors were given five months to complete policy personalisation and implementation. This included up to two months to personalise the physical activity policy template by selecting a minimum of five of 25 practices they would prioritise during the following three- to five-month implementation period. Directors were asked to return their physical activity policy via email to the research team for review and approval, conducted independently by two project officers. Once approved, services were asked to start implementing their policy and were provided with the remaining four implementation support strategies. Wait-listed comparison services were asked to continue their usual physical activity practices.

### Data collection

Baseline data collection (director and educator surveys) was conducted from January to June 2021 using online and paper surveys. Post-intervention data collection (director and educator surveys) was conducted from September 2021, and, despite attempts to complete this during 2021, continued until March 2022.

#### Primary effectiveness outcomes: changes in educator-reported time provided for children’s physical activity

At baseline and post-intervention, educators reported the amount of time provided daily for indoor and outdoor physical activity (two items) on seven-point ordinal scales (range ‘ < 30 min’- ‘180 + minutes’) and the amount of time provided daily for energetic play (one item) on a five-point ordinal scale (range ‘ < 15 min’- ‘60 + minutes’). Responses recorded for indoor and outdoor physical activity were summed using the minimum value of the response option range to provide a measure of total physical activity minutes per day. Total physical activity and energetic play were dichotomised based on the Play Active physical activity policy recommendations of: (i) ≥ 180 min of daily physical activity; (ii) ≥ 30 min of daily energetic play; and (iii) ≥ 180 min of physical activity and ≥ 30 min of energetic play per day. Items were based on established, validated instruments (i.e., Nutrition and Physical Activity Self-Assessment for Child Care [28] and Environment and Policy Assessment and Observation – Self Report [29]), and modified for the Australian ECEC context with acceptable test–retest reliability [30].

#### Secondary implementation outcomes: changes in educator- and director-reported uptake of physical activity policy practices

Educator-reported physical activity practice uptake was assessed in the baseline and post-intervention educator surveys through 21 items corresponding to the 15 educator-specific physical activity-related practices outlined in the policy template (Additional File 3). Twenty of these practice items were measured on six-point ordinal scales (range ‘never’- ‘always’), with the remaining item (frequency of providing outdoor play) measured using a six-point scale (range ‘zero times per day’- ‘five or more times per day’). Item responses of ‘always’ (or ‘never’ for negatively framed items) and ‘five or more times per day’ were summed to provide a total count of practice uptake. Items were based on established, validated instruments (Environment and Policy Assessment and Observation – Self Report [29]).

Director-reported physical activity practice uptake was assessed in intervention services immediately prior to policy implementation and at post-intervention through 25 items corresponding to the 25 policy practices in the policy template. All items were reported on a seven-point scale (range ‘never to be considered’- ‘longstanding practice’). Practice uptake was operationalised as the practice being ‘fully in place’ or ‘longstanding practice’, with in place practices summed to provide a total count. This process was repeated for the subset of seven high impact and low effort practices and the practices selected by services as priorities for implementation.

#### Process evaluation outcomes (intervention services only)

Fidelity of the six implementation support strategies was defined as whether the strategies were provided to services as intended [31]. Implementation support strategy reach, defined as uptake of the intervention within services [31], was obtained through project records and educator and director surveys (see survey items in Additional File 4). Professional development training reach was obtained through website analytics from the two professional development providers to identify individual educator (Provider 1) and service (Provider 2) completion. Completion of Provider 1 training was aggregated to service-level; a service was considered to have completed the professional development if at least one educator within the service completed the training. Play Active acceptability was assessed through five-point Likert scales measuring satisfaction and usefulness in the post-intervention educator and/or director surveys. Awareness of Play Active content, specifically the policy recommendation statements, was assessed in the post-intervention educator survey using ten true–false statements; correct responses were summed.

#### ECEC service and educator characteristics

Data available from ACECQA [32] were used to obtain the service size (number of approved places) and whether the service was a single- or multi-service provider. Service postcodes were matched to Australian Bureau of Statistics’ Socio-Economic Indexes for Areas data to obtain the relative disadvantage of service locations using the Index of Relative Socio-economic Disadvantage [33]. Educator characteristics included gender, year of birth, age/s of children they care for, usual hours of work in their ECEC room, and highest level of education.

### Analysis

Service- and educator-level characteristics were summarised by group. Differences between groups were tested for statistical significance using chi-square tests for categorical variables, t-tests for normally distributed continuous variables, and Wilcoxon tests for ordinal and skewed continuous variables.

The primary effectiveness outcomes were analysed using generalised linear mixed effects models (GLMM) and included fixed effects for group (intervention vs. wait-listed comparison), time (post-intervention vs. baseline), and time-by-group interaction; random intercept effects for individual educators nested within services; and an exchangeable correlation structure. Additional fixed effects for educator age and highest level of education were included in adjusted models. Since data collection spanned several months, we also considered the effect of total rainfall and maximum temperature; these were not significant confounders in any models and were thus not included in the present analyses. The primary outcomes were analysed as dichotomous variables for meeting the policy recommendations and as ordinal variables for minutes of total physical activity and energetic play. Since GLMMs allow for missing data [34], all educators were included in the analysis if they provided outcome data for at least one of the two timepoints. All primary outcomes were analysed at the educator-level following intention-to-treat protocols and each ECEC service represented a unique cluster. Analysis of director- and educator-reported uptake of policy practices followed the same approach. Data preparation and cleaning were carried out using SAS 9.4 and GLMMs were carried out in Stata 17 using the melogisitc command for dichotomous outcomes, meologistic command for ordinal outcomes, and mepoisson command for count data (uptake of practices). Process outcomes for intervention services were analysed descriptively and, for outcomes at the educator-level, adjusted for service-level clustering. Sensitivity analyses methods and results are provided in Additional File 6.

## Results

### ECEC service and educator characteristics

**Fig. 1:**
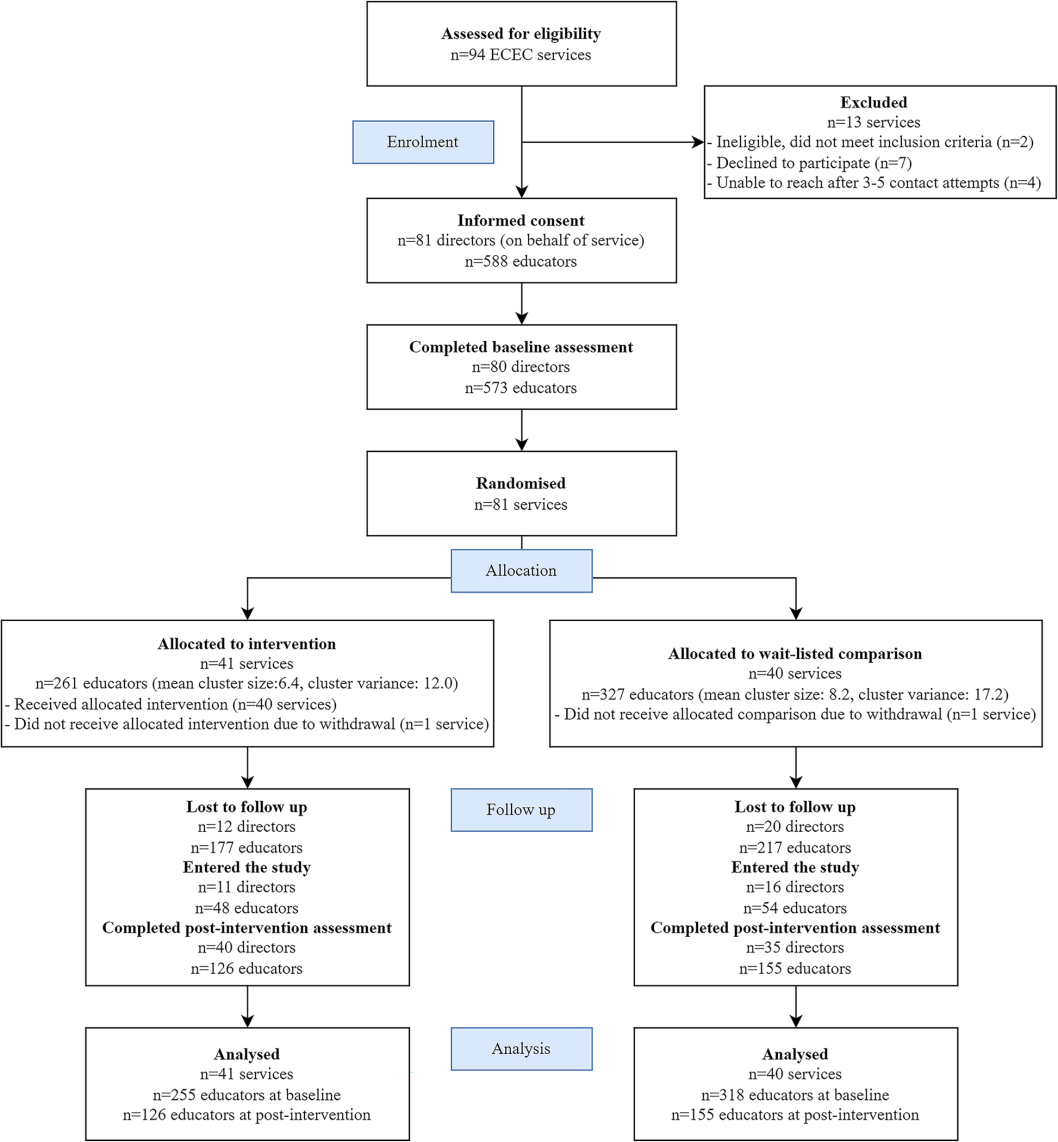
Flow of clusters and participants through each stage of the pragmatic cluster randomised trial

Figure 1 presents a flowchart of eligible and participating ECEC services and educators during the trial. On average, trial services had 73 approved places for children and almost all services were part of a larger provider (87.7%, Table 2). Wait-listed comparison services had on average 11 more approved places than intervention services (*p* = 0.027). About one-third of services were in the least disadvantaged socio-economic quintile.

**Table 2:** Baseline ECEC service characteristics by experimental group

		Total (*N* = 81)		Wait-listed comparison group (*N* = 40)		Intervention group (*N* = 41)		p-value*
Mean approved places		73.1		78.5		67.9		0.027
		N	%	N	%	N	%	*p*-value*
Provider type	Individual service	10	12.3	5	12.5	5	12.2	0.967
	Part of larger provider	71	87.7	35	87.5	36	87.8	
SEIFA quintile^a^	1 Most disadvantaged	5	6.2	2	5.0	3	7.3	0.788
	2	16	19.8	11	27.5	5	12.2	
	3	13	16.0	6	15.0	7	17.1	
	4	18	22.2	5	12.5	13	31.7	
	5 Least disadvantaged	29	35.8	16	40.0	13	31.7	

*Notes*:

^*^Statistically significant differences between wait-listed comparison group and intervention group (*p* < 0.05) in bold font

^a^Socio-Economic Indexes for Australia, Index of Relative Socio-Economic Disadvantage 2016 for Western Australia were matched to service postcodes

Almost all educators were female and half had a trade or diploma education level (Table 3). Across both groups, the length of time educators had worked in the ECEC sector (approximately six years) was substantially greater than the amount of time they had worked within their current service (approximately 1 to 2 years). Educators in the wait-listed comparison group had worked at their current service for significantly longer than educators in the intervention group at baseline (median 24 vs.14 months respectively; p = 0.001). Educator socio-demographic factors were similar between baseline and post-intervention. Directors returned their personalised policies in a median of 27 days (range 7–62) and had a median of 123 days to implement their selected policy practices (range 88–143) before post-intervention data collection.

**Table 3:** Educator characteristics by experimental group at baseline and post-intervention

	Wait-listed comparison group		Intervention group		*p*-value*	*p*-value^
	Baseline	Post-intervention	Baseline	Post-intervention		
	*N* = 318	*N* = 155	*N* = 255	*N* = 126		
Age	*N* = 313	*N* = 150	*N* = 255	*N* = 123	0.554	0.822
Years – median (IQR)	34.0 (15.0)	34.0 (15.0)	32.0 (14.0)	35.0 (18.0)		
Length of time worked in childcare sector	*N* = 317	*N* = 155	*N* = 249	*N* = 125	0.320	0.718
Months – median (IQR)	73.0 (104.0)	83.0 (115.0)	71.0 (91.0)	80.0 (93.0)		
Length of time worked in current service	*N* = 314	*N* = 154	*N* = 253	*N* = 124	**0.001**	**0.002**
Months – median (IQR)	24.0 (56.0)	31.5 (64.0)	14.0 (30.0)	21.0 (35.0)		
Usual time spent working in room in service	*N* = 309	*N* = 153	*N* = 250	*N* = 121	** < 0.001**	**0.050**
Hours per week – median (IQR)	37.5 (8.0)	37.5 (8.0)	35.0 (12.5)	36.0 (12.0)		
Gender	*N* = 318	*N* = 149	*N* = 254	*N* = 124	0.495	0.896
Female – n (%)	315 (99.1)	148 (99.3)	250 (98.4)	123 (99.2)		
Highest schooling completed	*N* = 316	*N* = 150	*N* = 254	*N* = 124	0.551	0.193
Year 12 or lower – n (%)	70 (22.2)	40 (26.7)	53 (20.9)	34 (27.4)		
Trade or diploma – n (%)	155 (49.1)	66 (44.0)	136 (53.5)	65 (52.4)		
University degree – n (%)	91 (28.8)	44 (29.3)	65 (25.6)	25 (20.2)		
Work in room with infants (aged under 1 years)	*N* = 318	*N* = 155	*N* = 255	*N* = 126	0.535	0.340
Yes – n (%)	80 (25.2)	40 (25.8)	70 (27.5)	39 (31.0)		
Work in room with toddlers (aged 1–2 years)	*N* = 318	*N* = 155	*N* = 255	*N* = 126	0.411	0.510
Yes – n (%)	221 (69.5)	100 (64.5)	169 (66.3)	86 (68.3)		
Work in room with kindergarten children (aged 3–5 years)	*N* = 318	*N* = 155	*N* = 255	*N* = 126	0.634	0.531
Yes – n (%)	172 (54.1)	89 (57.4)	143 (56.1)	77 (61.1)		
Received physical activity PD in the last two years^a^	*N* = 283	N/A	*N* = 236	N/A	0.274	N/A
Yes – n (%)	204 (72.1)		159 (67.7)			

*Notes*:

^*^Statistically significant differences between wait-listed comparison group and intervention group at baseline (*p* < 0.05) in bold font

^Statistically significant differences between wait-listed comparison group and intervention group at post-intervention (*p* < 0.05) in bold font

^a^Received professional development on recommended amounts of daily physical activity and energetic play for young children or encouraging physical activity and energetic play in young children at least once in the last two years. Measured only at baseline since providing professional development was an intervention implementation strategy

### Primary effectiveness outcomes: changes in educator-reported time provided for children’ physical activity

At baseline, 72.1% of wait-listed educators and 64.5% of intervention educators reported they provided at least 180 min of total physical activity to children (Table 4). Additionally, 84.2% of wait-listed comparison and 79.6% of intervention educators reported they provided children aged 3–5 with at least 30 min of energetic play each day. Over half of educators at baseline reported meeting both policy recommendations for physical activity provision (64.4% wait-listed comparison, 57.1% intervention). There were no significant group, time, or group-by-time effects for meeting physical activity policy recommendations or for daily time provided for total physical activity or energetic play (Table 5; unadjusted models can be found in Additional File 4).

**Table 4:** Descriptive statistics for educator-reported effectiveness and implementation outcome, by experimental group

	Wait-listed comparison group		Intervention group	
**Effectiveness outcomes**	Baseline	Post-intervention	Baseline	Post-intervention
Meets policy recommendation of providing 180 + mins/day of physical activity in young children (*N* = 655)	*N* = 308	*N* = 147	*N* = 248	*N* = 123
Yes – n (%)	222 (72.1)	108 (73.5)	160 (64.5)	76 (61.8)
Meets policy recommendation of providing 30 + mins/day of energetic play in kindergarten children (*N* = 646)	*N* = 303	*N* = 144	*N* = 245	*N* = 121
Yes – n (%)	255 (84.2)	129 (89.6)	195 (79.6)	102 (84.3)
Meets policy recommendation of providing 180 + mins/day of physical activity and 30 + mins/day of energetic play in kindergarten children (*N* = 646)	*N* = 303	*N* = 144	*N* = 245	*N* = 121
Yes – n (%)	195 (64.4)	96 (66.7)	140 (57.1)	71 (58.7)
Total time provided for indoor and outdoor physical activity (*N* = 655)	*N* = 308	*N* = 147	*N* = 248	*N* = 123
Median (IQR)	207.9 (140.4)	214.2 (161.0)	186.2 (156.8)	175.6 (127.4)
0 min/day – n (%)	0 (0.0)	0 (0.0)	4 (1.6)	0 (0.0)
30 min/day – n (%)	5 (1.6)	2 (1.4)	6 (2.4)	3 (2.4)
60 min/day – n (%)	20 (6.5)	3 (2.0)	20 (8.1)	6 (4.9)
90 min/day – n (%)	13 (4.2)	8 (5.4)	15 (6.0)	14 (11.4)
120 min/day – n (%)	18 (5.8)	14 (9.5)	22 (8.9)	17 (13.8)
150 min/day – n (%)	30 (9.7)	12 (8.2)	21 (8.5)	7 (5.7)
180 min/day – n (%)	29 (9.4)	17 (11.6)	30 (12.1)	17 (13.8)
210 min/day – n (%)	42 (13.6)	15 (10.2)	29 (11.7)	17 (13.8)
240 min/day – n (%)	39 (12.7)	18 (12.2)	20 (8.1)	16 (13.0)
270 min/day – n (%)	24 (7.8)	7 (4.8)	19 (7.7)	4 (3.3)
300 min/day – n (%)	29 (9.4)	13 (8.8)	14 (5.6)	8 (6.5)
330 min/day – n (%)	11 (3.6)	7 (4.8)	6 (2.4)	2 (1.6)
360 min/day – n (%)	48 (15.6)	31 (21.1)	42 (16.9)	12 (9.8)
Time provided for energetic play (*N* = 646)	*N* = 303	*N* = 144	*N* = 245	*N* = 121
< 15 min/day – n (%)	2 (0.7)	3 (2.1)	9 (3.7)	3 (2.5)
15–29 min/day – n (%)	46 (15.2)	12 (8.3)	41 (16.7)	16 (13.2)
30–44 min/day – n (%)	55 (18.2)	28 (19.4)	56 (22.9)	31 (25.6)
45–59 min/day – n (%)	51 (16.8)	29 (20.1)	36 (14.7)	16 (13.2)
60 + mins/day – n (%)	149 (49.2)	72 (50.0)	103 (42.0)	55 (45.5)
**Implementation outcome**				
Uptake of practices (total count)^a^ (N = 657)	*N = *308	*N = *151	*N* = 251	*N = *125
Median (IQR)	5.1 (6.8)	5.7 (6.8)	5.6 (6.4)	6.3 (8.1)

*Notes*: Percentages are adjusted for ECEC clustering

^a^Total count of practices consists of 21 physical activity practices corresponding to 15 of the practices outlined in the policy template

**Table 5:** Changes in adjusted educator-reported effectiveness and implementation outcomes

	Experimental group			Time			Experimental group by time interaction			ICC
**Effectiveness outcomes**	B (95% CI)	OR (95% CI)	*P*-value*	B (95% CI)	OR (95% CI)	*P*-value*	B (95% CI)	OR (95% CI)	*P*-value*	
Meets policy recommendation of providing 180 + mins/day of physical activity in young children (*N* = 643)	-0.4 (-1.0–0.2)	0.7 (0.4–1.2)	0.168	0.0 (-0.6–0.6)	1.0 (0.6–1.9)	0.917	-0.2 (-1.0–0.7)	0.8 (0.4–2.0)	0.694	0.08
Meets policy recommendation of providing 30 + mins/day of energetic play in kindergarten children (*N* = 634)	-0.4 (-1.1–0.3)	0.6 (0.3–1.3)	0.223	0.8 (-0.1–1.7)	2.3 (0.9–5.7)	0.068	-0.3 (-1.5–0.9)	0.7 (0.2–2.4)	0.613	0.00
Meets policy recommendation of providing 180 + mins/day of physical activity and 30 + mins/day of energetic play in kindergarten children (*N* = 634)	-0.4 (-0.9–0.2)	0.7 (0.4–1.2)	0.174	0.1 (-0.4–0.7)	1.1 (0.7–1.9)	0.633	0.0 (-0.7–0.8)	1.0 (0.5–2.2)	0.943	0.05
Total time provided for physical activity (*N* = 643)	-0.3 (-0.8–0.2)	0.7 (0.5–1.2)	0.222	0.2 (-0.2–0.6)	1.2 (0.8–1.9)	0.305	-0.5 (-1.1–0.1)	0.6 (0.3–1.1)	0.101	0.08
Time provided for energetic play (*N* = 634)	-0.4 (-0.9–0.1)	0.7 (0.4–1.1)	0.104	0.0 (-0.5–0.5)	1.0 (0.6–1.7)	0.896	0.2 (-0.5–0.9)	1.2 (0.6–2.4)	0.649	0.03
**Implementation outcome**	B (95% CI)	IRR (95% CI)	*P*-value*	B (95% CI)	IRR (95% CI)	*P*-value*	B (95% CI)	IRR (95% CI)	*P*-value*	ICC
Uptake of practices (total count)^a^ (*N* = 645)	0.0 (-0.1–0.2)	1.0 (0.9–1.2)	0.419	0.1 (-0.0–0.2)	1.1 (1.0–1.2)	0.137	-0.1 (-0.2–0.1)	0.9 (0.8–1.1)	0.384	N/A

*Notes*: Models are adjusted for educator age and education

*ICC* intraclass correlation at the service level, *N/A* not available for poisson regression

^*^Statistically significant coefficients (*p* < 0.05) in bold font

^a^Total count of practices consists of 21 physical activity practices corresponding to 15 of the practices outlined in the policy template

### Secondary implementation outcomes: changes in educator- and director-reported uptake of policy physical activity practices

At baseline, educators in the wait-listed comparison group reported ‘always’ using a median of 5.1 of a possible 21 physical activity-related practices, and intervention group educators reported ‘always’ using a median of 5.6 practices (Table 4). The time-by-group interaction was not significant (Table 5).

Intervention directors reported a median of 15.5 of 25 practices in the physical activity policy were in place in their service at baseline. At post-intervention, this had significantly increased to a median of 17.0 (IRR = 1.1, *p* = 0.034) (Table 6). Of the seven high impact and low effort policy practices, a median of 4.0 were in place at baseline which increased to a median of 6.0 at post-intervention but this change was not significant. There were no changes in the uptake of practices directors had selected to focus on during the implementation period (Table 6).

**Table 6:** Changes in director-reported implementation outcomes for intervention group only

	Baseline	Post-intervention	Unadjusted B (95% CI)	Unadjusted IRR (95% CI)	Unadjusted *P*-value*
Uptake of practices (total count)^a^ (N = 40)	*N* = 36	*N* = 40	0.1 (0.0–0.2)	1.1 (1.0–1.3)	**0.034**
Median (IQR)	15.5 (12.0)	17.0 (11.5)			
Uptake of high impact and low effort practices (total count)^b^ (N = 40)	*N* = 36	*N* = 40	0.2 (-0.0–0.4)	1.2 (1.0–1.5)	0.052
Median (IQR)	4.0 (3.0)	6.0 (3.0)			
	Baseline	Post-intervention	Unadjusted B (95% CI)		Unadjusted *P*-value*
Uptake of practices (out of selected practices) (N = 40)	*N* = 34	*N* = 40	2.7 (-0.9–6.2)		0.139
Proportion	37.5 (41.7)	32.4 (43.1)			
Uptake of high impact and low effort practices (out of selected high impact and low effort practices) (N = 40)	*N* = 34	*N* = 40	4.1 (-1.1–9.3)		0.123
Proportion	53.6 (66.7)	53.6 (63.3)			

*Notes*:

^*^Statistically significant differences between post-intervention and baseline (*p* < 0.05) in bold font

^a^Total count of practices consists of 25 policy practices being ‘fully in place’ or ‘longstanding practice’

^b^Total count of the seven high impact and low effort policy practices being ‘fully in place’ or ‘longstanding practice’

### Process evaluation outcomes

Fidelity of the six implementation support strategies was high. Forty intervention services were provided with the physical activity policy template and all services were provided with the six implementation support strategies (two services were not contacted for a reminder to return their policy as they completed this prior to the first contact).

Reach of the policy, policy personalisation, and policy review within services were high, with 74.5% of educators knowing their service had a policy, 98.7% of educators knowing where to find their policy, 100% of intervention services personalising their policy, and 100% of services meeting minimum policy criteria. Use of the energetic play assessment tool was also high, with 80.0% of services using it at baseline and 75.0% at post-intervention. However, only about half (53.6%) of educators reported using the resource guide, and of these, most (57.9%) used it less than weekly. Of the educators who reported using the resource guide, 68% found it to be very useful or extremely useful. In addition, of the 520 intervention educators signed up to the professional development website (Provider 1), 11.0% enrolled in the training and only 5.4% completed it (website analytics). Although, 39.1% of educators reported at post-intervention they had used the online training. One-third of intervention services had at least one educator complete the professional development training. No services completed the service-level professional development (Provider 2). Most services (87.5%) were able to be contacted at the mid-point of implementation to discuss their progress.

Most directors agreed or strongly agreed educators in their service thought Play Active was useful (75.0%), were willing to engage with Play Active (85.0%), understood the Play Active policy recommendations (82.5%), were confident to apply the recommendations (75.0%), and were enthusiastic about Play Active (62.5%). For educators who reported using the resource guide or online professional development, the majority rated the materials useful.

Overall satisfaction and acceptability of the Play Active policy intervention and implementation support was generally high at post-intervention: 82.9% of educators and 77.5% of directors were satisfied or very satisfied with Play Active. Though 58% of directors found Play Active very useful or extremely useful. Finally, intervention educator awareness of the Play Active policy recommendations was very high, with educators correctly answering a median 9.0 out of 10 statements (IQR = 2.0, *N* = 96).

### Harms

No harms or unintended effects were reported during the trial.

## Discussion

This study reports the effects of the Play Active ECEC policy intervention on the daily time educators provide for children to be physically active, and implementation and process evaluation outcomes. Educator-reported time for children’s daily physical activity did not significantly change. However, there was a significant increase in director-reported uptake of physical activity policy practices and a non-significant 33% increase in director-reported uptake of high impact and low effort policy practices. The Play Active intervention had high fidelity of implementation support, excellent reach for four of the six implementation support strategies, and high acceptability and awareness among ECEC staff. However, reach of two implementation support strategies (resource guide and professional development) was poor.

Supportive physical activity policy environments in ECEC services can improve educator’s physical activity practices and children’s physical activity levels [11–14]. Prior research shows just 16% of Western Australian ECEC services mention physical activity in their policies, with even fewer having a specific physical activity policy [17]. In the present study, 100% of ECEC services randomised to the Play Active intervention had a comprehensive evidence-informed physical activity policy following participation in the trial. In addition, there was a significant increase in director-reported uptake of physical activity policy practices, suggesting positive changes along the policy implementation to behaviour change pathway. Fidelity, reach, acceptability, and awareness of Play Active’s physical activity policy were all high, suggesting the Play Active policy intervention is feasible and appropriate for use in ECEC. These findings may be particularly relevant for supporting Australian ECEC services to achieve the National Quality Standard 2.1 related to promoting children’s physical activity.

However, despite Play Active being well implemented and resulting in significant increases in director-reported physical activity practice uptake, there were no corresponding changes in educator-reported uptake of physical activity practices nor educator-reported time provided for physical activity. It is possible the difference in policy practice uptake reported by directors and educators means changes in physical activity practices were only observable at the whole of service level, or there was a disparity between what directors perceived was occurring in their service and educators self-reported practice.

The lack of significant change in educator-reported physical activity provision and practice uptake may be due in part to the relatively short policy intervention implementation period. A recent umbrella review of physical activity interventions in ECEC settings found that interventions ranged from two days to two years [35]. For physical activity policy interventions in ECEC services, most have an intervention duration of longer than six months [14]. In comparison, the current trial involved a three-to-five-month policy implementation period, which was based on ECEC sector advice to complete data collection and implementation within a calendar year. Settings-based physical activity policy interventions require longer implementation periods because they target multiple levels, including service providers, directors, educators, parents, and ECEC physical environments. They also thus require multi-level strategies which each have their own unique but related implementation barriers and enablers [14]. Since practice uptake changes were apparent in this study at the service level, longer implementation and follow-up may result in observed educator-level changes. However, longer implementation periods for physical activity policy-based interventions in ECEC may be inhibited by the annual progression of large groups of children from ECEC to full-time school and staff turnover, both of which will add challenges to longer term data collection. Despite the challenges of longer-term implementation and follow up, multi-year studies will be required to measure long term changes in educator provided time for physical activity and children’s physical activity levels.

Overall, few educators engaged with the free or low-cost professional development training and only half used the Play Active resource guide, meaning there was little opportunity for educators to improve their physical activity-related knowledge and skills. Reasons for the low uptake of these two implementation support strategies are likely related to the limited time educators in general have for professional development, for example lack of time needed to complete training modules or read resource material content [14]. Furthermore, the impact of the COVID-19 pandemic added further pressure to an already overworked and underpaid workforce [36, 37], meaning that taking on initiatives such as a new physical activity policy and spending paid time on professional development during work hours perhaps had even less priority than usual. Also, the Play Active resource guide was distributed at a service level, with one hard copy sent to each service. This may have contributed to only about half of educators reporting its use, as some educators were unaware of its existence or where it was located in their service. While improved access to the resource guide may in turn have improved its use, it is also possible that frequency of use was correlated with how often a service undertakes its curriculum programming. This may explain why the resource guide was only used less than weekly by most educators. Since research has highlighted the importance of professional development for improving educator’s physical activity practices [38], further research is needed to identify and overcome ECEC barriers to using training and resources.

Future ECEC physical activity policy research should include longer-term outcome and implementation evaluation across all levels of implementation to capture where, when, and how practice changes may be occurring. Furthermore, future ECEC policy interventions could include additional implementation support strategies, including audit, feedback, and providing prompts [14], which could improve the effectiveness of physical activity policy interventions. Future implementation strategies may also include engaging parents and obtaining a commitment of resources and funding, methods that have been suggested by ECEC educators as important for implementing physical activity policies [20].

### Strengths

To-date, a small body of research has examined the effects of physical activity policy interventions implemented in ECEC services [14, 39]. Few of these studies have been in Australia, and prior to this study, none in Western Australia. Compared to most studies, Play Active recruited a larger number of ECEC services to test the effectiveness of the policy intervention using a pragmatic trial. In addition, this study reported a range of primary (i.e., educator self-reported physical activity provided for children) and secondary (i.e., implementation and process) outcomes to better understand the effects of the intervention and implementation support strategies; most previous studies have reported only primary outcomes and intervention reach or acceptability [35, 40].

Play Active employed an evidence-informed approach to co-design the intervention with consumers (e.g., parents and ECEC educators, directors, and service providers) and a partner advisory group [19, 22, 38]. In addition, Play Active had a strong theoretical approach to act across multi-level factors in the behaviour change pathway (i.e., improvement in educator knowledge, skills, and self-efficacy improvement in short term physical activity practices increased time provided for children’s daily physical activity) and worked closely with consumers and partners to address policy implementation barriers. Furthermore, the intervention was developed with scale-up in mind meaning most implementation support strategies are suitable for use with a large number of ECEC services following fidelity-consistent adaptations [41]. Combined, these factors likely contributed to the successful implementation and high feasibility, reach, and acceptability of Play Active.

### Limitations

As previously noted, the Play Active policy intervention was limited by a short implementation period and lack of educator use of the resource guide and online professional development. Additional limitations relate to the self-selected ECEC sample, measurement methods, and broader challenges within the ECEC sector. The self-selection of services into the trial could have introduced some bias as these services may have been more likely to implement the policy and/or report more desirable physical activity outcomes. The high proportion of educators at baseline reporting they were providing policy-recommended amounts of physical activity suggests this may have been the case, however, it may also indicate the intervention would have greater effect among ECEC services not self-selecting into a trial, including services located in regional, remote, and disadvantaged areas.

While based on established instruments [28, 29] validated in the Australian ECEC setting [30], the use of ordinal scales as response options for educator-reported time provided for physical activity and energetic play may have reduced the ability to detect small changes which may have contributed to null findings. In addition, these measures do not provide information on the types and intensities of physical activity children are achieving within this provided time. Device-based measures of physical activity, such as accelerometry, are sensitive to change and provide data on physical activity intensity but were not included due to the logistical and resourcing limitations of undertaking accelerometry with such a large number of ECEC services and children. Future research should consider the interplay between the educator-child physical activity behaviour change pathway, ECEC physical activity policy implementation period, and measurable changes in children's physical activity levels at ECEC.

Challenges within the Australian ECEC sector were exacerbated during the COVID-19 pandemic and in turn impacted the study. During the pandemic, high staff turnover rates and understaffing were common in the sector [36], which meant that ECEC staff regularly moved services. We observed one-quarter of all intervention services had director changes during the study, despite attempts to minimise this by making it part of the eligibility criteria. In addition, educators typically worked at their current service for less than two years and about 70% of educators who completed the baseline survey were lost to follow up. The staffing concerns and additional health-related protocols (i.e., extra hand washing, surface disinfection, visitor requirements etc.) during the COVID-19 pandemic also led to excessive workloads, with educators having less time to provide high quality education and care as well as less time for programming [36]. These factors can significantly disrupt the usual practices of services and thus may have negatively impacted the uptake of the Play Active policy intervention. However, these COVID-related limitations also reflect the nature of conducting a pragmatic trial during a pandemic.

## Conclusion

Overall, educator-reported daily time provided for children to be physically active did not change as a result of the Play Active intervention. However, there was a significant increase in director-reported uptake of physical activity policy practices by intervention services. In addition, the intervention resulted in all services establishing an evidence-based physical activity policy. Play Active was feasible, had generally high reach, and was considered acceptable by ECEC service directors and educators. The short implementation period and contextual barriers beyond our control (i.e., COVID-19 and staff turnover) limited the ability of ECEC services to create systemic changes and for the evaluation measures to detect changes in daily time provided for children’s physical activity. Despite Play Active being a well implemented intervention, further research is needed to ascertain the longer-term effects of the intervention on educator behaviour and children’s physical activity. Future ECEC policy-based research should incorporate assessment over a longer implementation period, consider how to better engage educators to undertake relevant professional development and training, and more effectively address the multi-level barriers to physical activity policy implementation. Future research should also investigate the scalability potential of Play Active, given the positive physical activity practice change findings.

## Supplementary Information

**Additional file 1. **

**Additional file 2. **

**Additional file 3. **

**Additional file 4.**

**Additional file 5. **

**Additional file 6. **

## Data Availability

The datasets used and/or analysed during the current study are available from the corresponding author on reasonable request.

## Abbreviations

ACECQA: Australian Children’s Education & Care Quality Authority
CONSORT: Consolidated Standards of Reporting Trials
ECEC: Early childhood education and care
GLMM: Generalised linear mixed effects models
TIDieR: Template for Intervention Description and Replication
WHO: World Health Organization
