# Supplementary material for: Play Active physical activity policy intervention and implementation support in early childhood education and care: results from a pragmatic cluster randomised trial
Source: Int J Behav Nutr Phys Act. 2023 Apr 20;20:46. doi: 10.1186/s12966-023-01442-0 (PMC10118225; doi:10.1186/s12966-023-01442-0)
Supplement: Supplementary file 1 — Additional file 1. [file 12966_2023_1442_MOESM1_ESM.docx]

## Additional File 1

Additional Table 1. CONSORT checklist, cluster and pragmatic trial additions italicised.

| Section/Topic | Item No | Checklist item | Reported on page No |
| --- | --- | --- | --- |
| Title and abstract | | | |
|  | 1a | Identification as a *pragmatic cluster* randomised trial in the title | 1 |
|  | 1b | Structured summary of trial design, methods, results, and conclusions | 3 |
| Introduction | | | |
| Background and objectives | 2a | Scientific background and explanation of rationale.  *Rationale for using a cluster design.*  *Describe the health or health service problem that the intervention is intended to address and other interventions that may commonly be aimed at this problem* | 5-6 |
|  | 2b | Specific objectives or hypotheses.  *Whether the objectives pertain to the cluster or individual level, or both,* | 6 |
| Methods | | | |
| Trial design | 3a | Description of trial design (such as parallel, factorial) including allocation ratio.  *Definition of cluster and how design features apply to clusters.* | 7, 12 |
|  | 3b | Important changes to methods after trial commencement (such as eligibility criteria), with reasons | 7 |
| Participants | 4a | Eligibility criteria for participants.  *Eligibility criteria for clusters.*  *Eligibility criteria should be explicitly framed to show the degree to which they include typical participants and/or, where applicable, typical providers (eg, nurses), institutions (eg, hospitals), communities (or localities eg, towns) and settings of care (eg, different healthcare financing systems)* | 7 |
|  | 4b | Settings and locations where the data were collected | 7 |
| Interventions | 5 | The interventions for each group with sufficient details to allow replication, including how and when they were actually administered.  *Whether interventions pertain to cluster or individual level, or both.*  *Describe extra resources added to (or resources removed from) usual settings in order to implement intervention. Indicate if efforts were made to standardise the intervention or if the intervention and its delivery were allowed to vary between participants, practitioners, or study sites. Describe the comparator in similar detail to the intervention.* | 8-9, Table 1 p29-31 |
| Outcomes | 6a | Completely defined pre-specified primary and secondary outcome measures, including how and when they were assessed.  *Whether the outcome measures pertain to the cluster or individual level, or both.*  *Explain why the chosen outcomes and, when relevant, the length of follow-up are considered important to those who will use the results of the trial* | 9-11 |
|  | 6b | Any changes to trial outcomes after the trial commenced, with reasons | N/A |
| Sample size | 7a | How sample size was determined.  *Method of calculation, number of clusters, cluster size, intracluster correlation and indication of its uncertainty.*  *If calculated using the smallest difference considered important by the target decision maker audience (the minimally important difference) then report where this difference was obtained.* | 7 |
|  | 7b | When applicable, explanation of any interim analyses and stopping guidelines | N/A |
| Randomisation: |  |  |  |
| Sequence generation | 8a | Method used to generate the random allocation sequence | 8 |
|  | 8b | Type of randomisation; details of any restriction (such as blocking and block size).  *Details of stratification or matching if used.* | 8 |
| Allocation concealment mechanism | 9 | Mechanism used to implement the random allocation sequence (such as sequentially numbered containers), describing any steps taken to conceal the sequence until interventions were assigned.  *Specification that allocation based on clusters not individuals and whether allocation concealment (if any) was at the cluster or individual level, or both.* | 8 |
| Implementation | 10a | *Who generated the random allocation sequence, who enrolled clusters, and who assigned clusters to intervention* | 7, 8 |
|  | 10b | *Mechanism by which individual participants were included in clusters for the purposes of the trial (such as complete enumeration, random sampling* | 7 |
|  | 10c | *From whom consent was sought (representatives of the cluster, or individual cluster members, or both), and whether consent was sought before or after randomisation* | 7, 8 |
| Blinding | 11a | If done, who was blinded after assignment to interventions (for example, participants, care providers, those assessing outcomes) and how.  *If blinding was not done or not possible, explain why.* | 8 |
|  | 11b | If relevant, description of the similarity of interventions | N/A |
| Statistical methods | 12a | Statistical methods used to compare groups for primary and secondary outcomes.  *How clustering was taken into account.* | 11-12 |
|  | 12b | Methods for additional analyses, such as subgroup analyses and adjusted analyses | 47 |
| Results | | | |
| Participant flow (a diagram is strongly recommended) | 13a | For each group, the numbers of participants who were randomly assigned, received intended treatment, and were analysed for the primary outcome.  *For each group, the numbers of clusters that were randomly assigned, received intended treatment, and were analysed for the primary outcome.*  *The number of participants or units approached to take part in the trial, the number which were eligible, and reasons for non-participation should be reported.* | Figure 1 |
|  | 13b | For each group, losses and exclusions after randomisation, together with reasons.  *For each group, losses and exclusions for both clusters and individual cluster members.* | Figure 1 |
| Recruitment | 14a | Dates defining the periods of recruitment and follow-up | 9 |
|  | 14b | Why the trial ended or was stopped | N/A |
| Baseline data | 15 | A table showing baseline demographic and clinical characteristics for each group.  *Baseline characteristics for the individual and cluster levels as applicable for each group.* | Table 2 p31  Table 3 p 32 |
| Numbers analysed | 16 | For each group, number of participants (denominator) included in each analysis and whether the analysis was by original assigned groups.  *For each group, number of clusters included in each analysis.* | Figure 1  Table 4 p34  Table 5 p35 |
| Outcomes and estimation | 17a | For each primary and secondary outcome, results for each group, and the estimated effect size and its precision (such as 95% confidence interval).  *Results at the individual or cluster level as applicable and a coefficient of intracluster correlation (ICC or k) for each primary outcome.* | Table 5 p35 |
|  | 17b | For binary outcomes, presentation of both absolute and relative effect sizes is recommended | Table 5 p35 |
| Ancillary analyses | 18 | Results of any other analyses performed, including subgroup analyses and adjusted analyses, distinguishing pre-specified from exploratory | 47-50 |
| Harms | 19 | All important harms or unintended effects in each group | 15 |
| Discussion | | | |
| Limitations | 20 | Trial limitations, addressing sources of potential bias, imprecision, and, if relevant, multiplicity of analyses | 18 |
| Generalisability | 21 | Generalisability (external validity, applicability) of the trial findings.  *Generalisability to clusters and/or individual participants (as relevant).*  *Describe key aspects of the setting which determined the trial results. Discuss possible differences in other settings where clinical traditions, health service organisation, staffing, or resources may vary from those of the trial* | 15-17 |
| Interpretation | 22 | Interpretation consistent with results, balancing benefits and harms, and considering other relevant evidence | 15-17 |
| Other information | | | |
| Registration | 23 | Registration number and name of trial registry | 4 |
| Protocol | 24 | Where the full trial protocol can be accessed, if available | 6 |
| Funding | 25 | Sources of funding and other support (such as supply of drugs), role of funders | 21-22 |
