# Supplementary material for: Play Active physical activity policy intervention and implementation support in early childhood education and care: results from a pragmatic cluster randomised trial
Source: Int J Behav Nutr Phys Act. 2023 Apr 20;20:46. doi: 10.1186/s12966-023-01442-0 (PMC10118225; doi:10.1186/s12966-023-01442-0)
Supplement: Supplementary file 2 — Additional file 2. [file 12966_2023_1442_MOESM2_ESM.docx]

## Additional File 2

Additional Table 2. TIDieR checklist for describing the intervention

| Item number | Item | Where located | |
| --- | --- | --- | --- |
|  |  | Primary paper  (page or appendix  number) | Other^ (details) |
|  | BRIEF NAME |  |  |
| 1. | Provide the name or a phrase that describes the intervention. | 8 |  |
|  | WHY |  |  |
| 2. | Describe any rationale, theory, or goal of the elements essential to the intervention. | 8-9 | Nathan et al. 2022. |
|  | WHAT |  |  |
| 3. | Materials: Describe any physical or informational materials used in the intervention, including those provided to participants or used in intervention delivery or in training of intervention providers. Provide information on where the materials can be accessed (e.g. online appendix, URL). | 8-9, Table 1 p29-31 | Nathan et al. 2022. |
| 4. | Procedures: Describe each of the procedures, activities, and/or processes used in the intervention, including any enabling or support activities. | 8-9, Table 1 p29-31 | Nathan et al. 2022. |
|  | WHO PROVIDED |  |  |
| 5. | For each category of intervention provider (e.g. psychologist, nursing assistant), describe their expertise, background and any specific training given. | 8-9, Table 1 p29-31 | Nathan et al. 2022. |
|  | HOW |  |  |
| 6. | Describe the modes of delivery (e.g. face-to-face or by some other mechanism, such as internet or telephone) of the intervention and whether it was provided individually or in a group. | 8-9, Table 1 p29-31 | Nathan et al. 2022. |
|  | WHERE |  |  |
| 7. | Describe the type(s) of location(s) where the intervention occurred, including any necessary infrastructure or relevant features. | 7 | Nathan et al. 2022. |
|  | WHEN and HOW MUCH |  |  |
| 8. | Describe the number of times the intervention was delivered and over what period of time including the number of sessions, their schedule, and their duration, intensity or dose. | 8 |  |
|  | TAILORING |  |  |
| 9. | If the intervention was planned to be personalised, titrated or adapted, then describe what, why, when, and how. | 8-9, Table 1 p29-31 |  |
|  | MODIFICATIONS |  |  |
| 10.^ǂ^ | If the intervention was modified during the course of the study, describe the changes (what, why, when, and how). | N/A |  |
|  | HOW WELL |  |  |
| 11. | Planned: If intervention adherence or fidelity was assessed, describe how and by whom, and if any strategies were used to maintain or improve fidelity, describe them. | 10-11 | Nathan et al. 2022. |
| 12.^ǂ^ | Actual: If intervention adherence or fidelity was assessed, describe the extent to which the intervention was delivered as planned. | 14, Table 1 p29-31 |  |

Notes:

^Reference details: Nathan A, Adams E, Trost S, Cross D, Schipperijn J, McLaughlin M, et al. Evaluating the effectiveness of the Play Active policy intervention and implementation support in early childhood education and care: a pragmatic cluster randomised trial protocol. BMC Public Health. 2022;22(1):306-.
