# Supplementary material for: Play Active physical activity policy intervention and implementation support in early childhood education and care: results from a pragmatic cluster randomised trial
Source: Int J Behav Nutr Phys Act. 2023 Apr 20;20:46. doi: 10.1186/s12966-023-01442-0 (PMC10118225; doi:10.1186/s12966-023-01442-0)
Supplement: Supplementary file 3 — Additional file 3. [file 12966_2023_1442_MOESM3_ESM.docx]

## Additional File 3

Additional Table 3. Physical activity policy recommendations and practices and related educator survey items.

| Recommendation number | Policy Recommendation | Educator survey item/s relating to recommendation | |
| --- | --- | --- | --- |
| 1 | To meet the Australian 24-Hour Movement Guidelines for the Early Years, provide children with at least 180 minutes of physical activity daily, via a variety of physical activities spread throughout the day. More is better. | The amount of time I provide children for indoor physical activity each day is  The amount of time I provide children for outdoor physical activity each day is | |
| 2 | For kindergarten children, this will include at least 30 minutes of ‘energetic play’ each day at ECEC. More is better. | The amount of time I provide to children for energetic play each day is | |
| Practice number | Policy Practice | Educator survey item/s relating to practice | Response option used for practice uptake count |
| 1 | Ensure awareness and understanding of this Physical Activity Policy*** | N/A^1^ |  |
| 2 | Provide many daily opportunities for outdoor play time*** | I provide children with outdoor play time: | 5 or more times per day |
| 3 | Build physical activity and energetic play into everyday experiences | I make physical activity and energetic play part of children's everyday experiences | Always |
| 4 | Program a range of learning experiences which encourage and use physical activity and energetic play | I program a range of learning experiences which encourage and use physical activity and energetic play | Always |
| 5 | Provide opportunities for children to engage in discovery learning and discuss the importance of physical activity*** | I provide opportunities for children to engage in discovery learning about the importance of physical activity and energetic play | Always |
| 6 | Break up prolonged periods of sedentary behaviour e.g. sitting or standing for long periods or infants confined to high chairs or cots when not eating or sleeping*** | I use cots, car seats and high chairs for their primary purpose only (cots for sleeping, car seats for vehicle travel, and high chairs for eating) | Always |
| 7 | Limit the use of equipment, such as strollers, swings and bouncer seats/chairs, for holding infants while they are awake | N/A^2^ |  |
| 8 | Not use punitive measures such as withholding physical activity as punishment for managing challenging behaviours (e.g. seated time out); not use physical activity as punishment (e.g. star jumps)*** | I take away 5 or more minutes of active play time if children misbehave | Never |
| 9 | Include physical activity as part of the assessment of children’s physical and overall development | I include physical activity as part of the assessment of children's physical and overall development | Always |
| 10 | Ensure age- and developmentally appropriate, structured and unstructured physical activity is provided for each child | I provide age and developmentally appropriate structured physical activity for each child  I provide age and developmentally appropriate unstructured physical activity for each child | Always  Always |
| 11 | Act as positive role models. Demonstrate and participate in physical activity and energetic play with children | I avoid sitting while supervising outside play  I join children in physically active play  I join children in running and chasing games | Always  Always  Always |
| 12 | Take part in professional development programs to increase knowledge and skills around children’s physical activity | N/A^3^ |  |
| 13 | Provide opportunities for all children (including children with disabilities) to be physically active | I provide opportunities for all children (including children with disabilities) to be physically active | Always |
| 14 | Provide children who have disabilities with equipment that meets the current standards for accessible design, to encourage physical activity | N/A^1^ |  |
| 15 | Provide an outdoor environment with a plentiful variety of portable and fixed play equipment, a secure perimeter, shade, natural elements, open grassy areas, free running space, connected paths, varying surfaces, and more than the minimum outdoor space per child where possible | N/A^1^ |  |
| 16 | Provide an indoor environment with a plentiful variety of portable (and fixed) play equipment, natural elements, unobstructed space to be active, and more than the minimum indoor space per child where possible | N/A^1^ |  |
| 17 | Ensure adequate physical activity opportunities in poor weather, e.g. very high or low temperatures, storms, or when the UV index is above 8. Provide indoor physical activity alternatives where possible | I provide children with adequate physical activity in poor weather | Always |
| 18 | Wear comfortable and appropriate clothing and footwear that doesn’t limit children’s and educator’s ability to engage in physical activity*** | I wear clothing and footwear that allows me to actively participate in physical activity  I encourage children to wear clothing and footwear that allows them to actively participate in physical activity | Always  Always |
| 19 | Provide a copy of this Physical Activity Policy to all families upon orientation at the service | N/A^1^ |  |
| 20 | Talk with families about their children’s physical activity | I talk with parents about their child's physical activity | Always |
| 21 | Communicate regularly with families about physical activity experiences within our service | I communicate regularly with families about physical activity experiences within our service | Always |
| 22 | Encourage parents to create plenty of opportunities for their child to engage in physical activity and energetic play at home, and provide information which supports them in this | I encourage parents to create plenty of opportunities for their child to engage in physical activity and energetic play at home | Always |
| 23 | Provide families with opportunities to contribute to the review and development of this policy | N/A^1^ |  |
| 24 | This Physical Activity Policy is available to staff, families and visitors*** | N/A^1^ |  |
| 25 | All staff including management and educators, and parents, monitor and review the effectiveness of the policy and revise the policy when required (at least once every three years) by completing a policy review and accreditation via the Play Active Program. | N/A^1^ |  |

Notes:

*** High impact and low effort practices.

^1^ Practice not relevant to individual educators and so not measured in educator survey.

^2^ Relevant survey item was only asked of educators who worked with infants and so item not included in total practice count.

^3^ Relevant survey items only included in baseline survey since professional development was provided as part of the intervention; items therefore not able to be included in total practice count.
