# Supplementary material for: Play Active physical activity policy intervention and implementation support in early childhood education and care: results from a pragmatic cluster randomised trial
Source: Int J Behav Nutr Phys Act. 2023 Apr 20;20:46. doi: 10.1186/s12966-023-01442-0 (PMC10118225; doi:10.1186/s12966-023-01442-0)
Supplement: Supplementary file 4 — Additional file 4. [file 12966_2023_1442_MOESM4_ESM.docx]

## Additional File 4

Additional Table 4. Process measures included in educator and director post-intervention surveys.

| Process outcome | Respondent | Survey item | Survey response options |
| --- | --- | --- | --- |
| Reach | Educator | Does your service have a physical activity policy?  [If yes,] Do you know where to find it? | Yes, No, Unsure  Yes, No, Unsure |
|  | Educator | Did you use the Play Active Resource Guide?  [If yes,] How often did you use the Play Active Resource Guide? | Yes, No, Unsure  7-point scale from ‘Once’ to ‘More than once per day’ |
|  | Educator | Did you do any of the professional development training provided by [Provider 1]? | Yes, No, Unsure |
|  | Educator | Did you do any of the professional development training provided by [Provider 2]? | Yes, No, Unsure |
| Acceptability | Director and educator | What is your overall satisfaction with the Play Active Program? | 5-point scale from ‘Very dissatisfied’ to ‘Very satisfied’ |
|  | Director | How useful did you find the Play Active Program? | 5-point scale from ‘Not at all useful’ to ‘Extremely useful’ |
|  | Director | Educators think the Play Active Program is useful for increasing children's physical activity. | 5-point scale from ‘Strongly disagree’ to ‘Strongly agree’ |
|  | Director | Educators are willing to engage in the Play Active Program. | 5-point scale from ‘Strongly disagree’ to ‘Strongly agree’ |
|  | Director | Educators understand the physical activity recommendations in our Physical Activity Policy. | 5-point scale from ‘Strongly disagree’ to ‘Strongly agree’ |
|  | Director | Educators are confident to apply the physical activity recommendations in our Physical Activity Policy. | 5-point scale from ‘Strongly disagree’ to ‘Strongly agree’ |
|  | Director | Educators are enthusiastic about the Play Active Program. | 5-point scale from ‘Strongly disagree’ to ‘Strongly agree’ |
|  | Educator | How useful did you find the Play Active Resource Guide? | 5-point scale from ‘Not at all useful’ to ‘Extremely useful’ |
|  | Educator | How useful did you find the professional development training provided by [Provider 1]? | 5-point scale from ‘Not at all useful’ to ‘Extremely useful’ |
|  | Educator | How useful did you find the professional development training provided by [Provider 2]? | 5-point scale from ‘Not at all useful’ to ‘Extremely useful’ |
| Awareness | Educator | Children need no more than an hour of physical activity each day. | True, **False** |
|  | Educator | Children aged 3-5 should have at least 30 minutes of energetic play each day at ECEC. | **True**, False |
|  | Educator | Infants can be active through a variety of supervised, interactive floor-based play such as crawling and games. | **True**, False |
|  | Educator | Infants who are not yet mobile need at least 30 minutes of tummy time per day. | **True**, False |
|  | Educator | Children should not be confined in a stroller or highchair for more than an hour at a time. | **True**, False |
|  | Educator | It is okay for children to sit for any length of time. | True, **False** |
|  | Educator | Children should not be given sedentary screen time for purposes other than learning. | **True**, False |
|  | Educator | Infants should only use cots, car seats, and high chairs for their primary purpose (e.g. high chairs for eating). | **True**, False |
|  | Educator | Infants can be confined to strollers, swings, or bouncer seats for any length of time. | True, **False** |
|  | Educator | Infants should not be given any screen time. | **True**, False |

Notes. Bolded awareness survey item response options indicate the correct response.
