# Supplementary material for: Play Active physical activity policy intervention and implementation support in early childhood education and care: results from a pragmatic cluster randomised trial
Source: Int J Behav Nutr Phys Act. 2023 Apr 20;20:46. doi: 10.1186/s12966-023-01442-0 (PMC10118225; doi:10.1186/s12966-023-01442-0)
Supplement: Supplementary file 5 — Additional file 5. [file 12966_2023_1442_MOESM5_ESM.docx]

## Additional File 5

Additional Table 5. Changes in unadjusted educator-reported effectiveness and implementation outcomes.

|  | Experimental group | | Time | | Experimental group by time interaction | | ICC |
| --- | --- | --- | --- | --- | --- | --- | --- |
| **Effectiveness outcomes** | OR (95% CI) | P-value | OR (95% CI) | P-value | OR (95% CI) | P-value |  |
| Meets policy recommendation of providing 180+ mins/day of physical activity in young children | 0.6 (0.4-1.2) | 0.159 | 1.0 (0.6-1.8) | 0.972 | 0.8 (0.3-1.8) | 0.568 | 0.08 |
| Meets policy recommendation of providing 30+ mins/day of energetic play in kindergarten children | 0.6 (0.3-1.3) | 0.231 | 2.2 (0.8-5.9) | 0.120 | 0.8 (0.2-2.8) | 0.745 | 0.00 |
| Meets policy recommendation of providing 180+ mins/day of physical activity and 30+ mins/day of energetic play in kindergarten children | 0.7 (0.4-1.2) | 0.174 | 1.1 (0.6-1.8) | 0.802 | 1.0 (0.5-2.2) | 0.994 | 0.05 |
| Total time provided for physical activity | 0.7 (0.4-1.2) | 0.198 | 1.2 (0.8-1.9) | 0.286 | 0.6 (0.3-1.1) | 0.078 | 0.08 |
| Time provided for energetic play | 0.6 (0.4-1.1) | 0.087 | 1.0 (0.6-1.7) | 0.860 | 1.2 (0.6-2.4) | 0.650 | 0.03 |
| **Implementation outcome** | IRR (95% CI) | P-value* | IRR (95% CI) | P-value* | IRR (95% CI) | P-value* |  |
| Uptake of practices (total count)^1^ | 1.0 (0.9-1.2) | 0.554 | 1.1 (1.0-1.2) | 0.173 | 0.9 (0.8-1.1) | 0.314 | NA |

Notes:

^1^ Total count of practices consists of 21 physical activity practices corresponding to 15 of the practices outlined in the policy template.

ICC=intraclass correlation at the service level.

NA=not available for poisson regression.
