# Supplementary material for: Play Active physical activity policy intervention and implementation support in early childhood education and care: results from a pragmatic cluster randomised trial
Source: Int J Behav Nutr Phys Act. 2023 Apr 20;20:46. doi: 10.1186/s12966-023-01442-0 (PMC10118225; doi:10.1186/s12966-023-01442-0)
Supplement: Supplementary file 6 — Additional file 6. [file 12966_2023_1442_MOESM6_ESM.docx]

## Additional File 6

### Sensitivity analyses

Three sensitivity analyses were performed. The first included only educators who provided baseline and post-intervention data. These models also adjusted for time-varying covariates of length of time in sector, length of time in service, and usual hours of work. Since the physical activity recommendations are age-specific, the second sensitivity analyses included only educators who indicated they worked with children of the relevant age group (1 to 5 years for total physical activity, 3 to 5 years for energetic play). As these were potentially time-varying variables, the models only included educators who worked with the relevant ages at both baseline and at post-intervention. To examine the effect of varying reach of the implementation support strategies into services (notably for use of the resource guide and professional development) on the effectiveness of the intervention, a third sensitivity analysis was performed including only the intervention group and a variable for high vs. low reach of the implementation strategies. High reach was defined as service-level completion of the online professional development course and the educator self-reporting they had used the resource guide.

Sensitivity analyses including only educators with baseline and post-intervention data showed a negative interaction effect for total minutes of physical activity, but this was not seen for the proportion providing policy-recommended amounts of physical activity. There were no other notable sensitivity analyses findings.

Additional Table 6. Sensitivity analysis 1: Changes in outcomes among educators with baseline and post-intervention data.

|  | Experimental group | | | Time | | | Experimental group by time interaction | | | ICC |
| --- | --- | --- | --- | --- | --- | --- | --- | --- | --- | --- |
| **Effectiveness outcomes** | B (95% CI) | OR (95% CI) | P-value* | B (95% CI) | OR (95% CI) | P-value* | B (95% CI) | OR (95% CI) | P-value* |  |
| Meets policy recommendation of providing 180+ mins/day of physical activity in young children (N=331) | -0.9 (-2.0-0.1) | 0.4 (0.1-1.1) | 0.077 | -0.2 (-1.0-0.6) | 0.8 (0.4-1.8) | 0.620 | -0.2 (1.3-0.9) | 0.8 (0.3-2.5) | 0.727 | 0.01 |
| Meets policy recommendation of providing 30+ mins/day of energetic play in kindergarten children (N=323) | -0.5 (-1.8-0.8) | 0.6 (0.2-2.1) | 0.437 | 0.8 (-0.2-1.9) | 2.3 (0.8-6.5) | 0.113 | -0.5 (-1.9-1.0) | 0.6 (0.1-2.6) | 0.514 | 0.11 |
| Meets policy recommendation of providing 180+ mins/day of physical activity and 30+ mins/day of energetic play in kindergarten children (N=323) | -0.8 (-1.8-0.1) | 0.4 (0.2-1.2) | 0.095 | -0.1 (-0.8-0.7) | 0.9 (0.5-1.9) | 0.865 | -0.0 (-1.1-1.0) | 1.0 (0.3-2.8) | 0.951 | 0.10 |
| Total time provided for physical activity | -0.6 (-1.3-0.2) | 0.6 (0.3-1.2) | 0.128 | -0.2 (-0.4-0.7) | 1.2 (0.7-2.0) | 0.527 | -0.8 (-1.6- -0.0) | 0.4 (0.2-1.0) | **0.044** | 0.00 |
| Time provided for energetic play (N=323) | -1.1 (-2.0- -0.2) | 0.3 (0.1-0.8) | **0.019** | -0.2 (-0.9-0.4) | 0.8 (0.4-1.5) | 0.462 | 0.2 (-0.7-1.1) | 1.3 (0.5-3.1) | 0.625 | 0.00 |
| **Implementation outcome** | B (95% CI) | IRR (95% CI) | P-value* | B (95% CI) | IRR (95% CI) | P-value* | B (95% CI) | IRR (95% CI) | P-value* | ICC |
| Uptake of practices (total count)^1^ | 0.1 (-0.1-0.3) | 1.1 (0.9-1.4) | 0.334 | 0.1 (-0.1-0.2) | 1.1 (0.9-1.2) | 0.317 | -0.1 (-0.2-0.1) | 0.9 (0.8-1.1) | 0.326 | NA |

Notes: Models are adjusted for educator age, education, time working in the sector, time working at current service, and usual hours of work. Analysis sample includes only educators with baseline and post-intervention data for that outcome.

^1^ Total count of practices consists of 21 physical activity practices corresponding to 15 of the practices outlined in the policy template.

ICC=intraclass correlation at the service level.

NA=not available for poisson regression.

*Statistically significant coefficients (p<0.05) in bold font.

Additional Table 7. Sensitivity analysis 2: Changes in outcomes among educators who worked with the relevant aged children.

|  | Experimental group | | | Time | | | Experimental group by time interaction | | | ICC |
| --- | --- | --- | --- | --- | --- | --- | --- | --- | --- | --- |
| **Effectiveness outcomes** | B (95% CI) | OR (95% CI) | P-value* | B (95% CI) | OR (95% CI) | P-value* | B (95% CI) | OR (95% CI) | P-value* |  |
| Meets policy recommendation of providing 180+ mins/day of physical activity in young children (N=313)^1^ | -0.9 (-2.0-0.2) | 0.4 (0.1-1.2) | 0.098 | -0.1 (1.0-0.7) | 0.9 (0.4-2.0) | 0.729 | -0.3 (-1.5-0.9) | 0.7 (0.2-2.4) | 0.601 | 0.02 |
| Meets policy recommendation of providing 30+ mins/day of energetic play in kindergarten children (N=164)^2^ | 0.1 (-1.7-1.9) | 1.1 (0.2-6.9) | 0.930 | 1.0 (-0.4-2.5) | 2.8 (0.6-12.6) | 0.168 | -0.8 (-2.8-1.3) | 0.5 (0.1-3.7) | 0.477 | 0.00 |
| Meets policy recommendation of providing 180+ mins/day of physical activity and 30+ mins/day of energetic play in kindergarten children (N=164)^2^ | -0.4 (-1.7-0.9) | 0.7 (0.2-2.5) | 0.563 | 0.5 (-0.5-1.6) | 1.7 (0.6-4.8) | 0.334 | -1.1 (-2.6-0.5) | 0.3 (0.1-1.6) | 0.183 | 0.00 |
| Total time provided for physical activity (N=313)^1^ | -0.5 (-1.3-0.3) | 0.6 (0.3-1.4) | 0.224 | 0.3 (-0.3-0.8) | 1.3 (0.8-2.2) | 0.349 | -0.9 (-1.7- -0.1) | 0.4 (0.2-0.9) | **0.033** | 0.04 |
| Time provided for energetic play (N=164)^2^ | -0.2 (-1.5-1.2) | 0.8 (0.2-3.3) | 0.806 | 0.0 (-0.8-0.9) | 1.0 (0.4-2.5) | 0.932 | -0.0 (-1.3-1.3) | 1.0 (0.3-3.6) | 0.970 | 0.00 |

Notes: Models are adjusted for educator age, education, time working in the sector, time working at current service, and usual hours of work. Analysis sample includes only educators with baseline and post-intervention data for that outcome and who worked with the age group relevant to that policy recommendation at baseline and post-intervention.

^1^ Includes educators who taught 1–5-year-olds at baseline and at post-intervention.

^2^ Includes educators who taught 3–5-year-olds at baseline and at post-intervention.

ICC=intraclass correlation at the service level.

*Statistically significant coefficients (p<0.05) in bold font.

Additional Table 11. Sensitivity analysis 3: Changes in outcomes among high vs low reach intervention educators.

|  | Experimental group | | | Time | | | Experimental group by time interaction | | | ICC |
| --- | --- | --- | --- | --- | --- | --- | --- | --- | --- | --- |
| **Effectiveness outcomes** | B (95% CI) | OR (95% CI) | P-value* | B (95% CI) | OR (95% CI) | P-value* | B (95% CI) | OR (95% CI) | P-value* |  |
| Meets policy recommendation of providing 180+ mins/day of physical activity in young children (N=197) | 0.4 (-1.1-1.8) | 1.4 (0.3-6.3) | 0.632 | 0.0 (-0.8-0.8) | 1.0 (0.5-2.3) | 0.920 | -1.4 (-3.1-0.4) | 0.3 (0.0-1.4) | 0.120 | 0.05 |
| Meets policy recommendation of providing 30+ mins/day of energetic play in kindergarten children (N=195) | -1.3 (-3.5-0.9) | 0.3 (0.0-2.5) | 0.243 | 0.8 (-0.5-2.1) | 2.2 (0.6-8.0) | 0.213 | -0.3 (-2.5-2.0) | 0.8 (0.1-7.2) | 0.814 | 0.00 |
| Meets policy recommendation of providing 180+ mins/day of physical activity and 30+ mins/day of energetic play in kindergarten children (N=195) | -0.6 (-2.1-0.9) | 0.6 (0.1-2.5) | 0.439 | 0.2 (-0.7-1.0) | 1.2 (0.5-2.8) | 0.612 | -0.8 (-2.5-0.9) | 0.4 (0.1-2.5) | 0.354 | 0.00 |
| Total time provided for physical activity (N=197) | -0.1 (-1.3-1.1) | 0.9 (0.3-3.1) | 0.890 | -0.2 (-0.8-0.4) | 0.8 (0.4-1.5) | 0.457 | -1.2 (-2.5-0.2) | 0.3 (0.1-1.2) | 0.087 | 0.07 |
| Time provided for energetic play (N=195) | -1.0 (-2.4-0.4) | 0.4 (0.1-1.4) | 0.146 | 0.2 (-0.5-0.9) | 1.3 (0.6-2.5) | 0.531 | 0.1 (-1.3-1.4) | 1.1 (0.3-4.2) | 0.931 | 0.00 |
| **Implementation outcome** | B (95% CI) | IRR (95% CI) | P-value* | B (95% CI) | IRR (95% CI) | P-value* | B (95% CI) | IRR (95% CI) | P-value* | ICC |
| Uptake of practices (total count) (N=199)^1^ | 0.1 (-0.3-0.5) | 1.1 (0.8-1.6) | 0.640 | -0.1 (-0.2-0.1) | 0.9 (0.8-1.1) | 0.299 | 0.2 (-0.1-0.4) | 1.2 (0.9-1.5) | 0.255 | NA |

Notes: Experimental group is high reach vs. low reach, based on centre level PD completion and self-report use of resource guide. Models are adjusted for educator age and education.

^1^ Total count of practices consists of 21 physical activity practices corresponding to 15 of the practices outlined in the policy template.

ICC=intraclass correlation at the service level.

NA=not available for poisson regression.

*Statistically significant coefficients (p<0.05) in bold font.
